# Imaging deductive reasoning and the new paradigm

**DOI:** 10.3389/fnhum.2015.00101

**Published:** 2015-02-27

**Authors:** Mike Oaksford

**Affiliations:** Department of Psychological Sciences, Birkbeck College, University of LondonLondon, UK

**Keywords:** Marr’s levels, Bayesian inference, brain imaging, new paradigm

## Abstract

There has been a great expansion of research into human reasoning at all of Marr’s explanatory levels. There is a tendency for this work to progress within a level largely ignoring the others which can lead to slippage between levels (Chater et al., 2003). It is argued that recent brain imaging research on deductive reasoning—implementational level—has largely ignored the new paradigm in reasoning—computational level (Over, 2009). Consequently, recent imaging results are reviewed with the focus on how they relate to the new paradigm. The imaging results are drawn primarily from a recent meta-analysis by Prado et al. (2011) but further imaging results are also reviewed where relevant. Three main observations are made. First, the main function of the core brain region identified is most likely elaborative, defeasible reasoning not deductive reasoning. Second, the subtraction methodology and the meta-analytic approach may remove all traces of content specific System 1 processes thought to underpin much human reasoning. Third, interpreting the function of the brain regions activated by a task depends on theories of the function that a task engages. When there are multiple interpretations of that function, interpreting what an active brain region is doing is not clear cut. It is concluded that there is a need to more tightly connect brain activation to function, which could be achieved using formalized computational level models and a parametric variation approach.

This paper presents a focused review of the brain imaging results on deductive reasoning. The focus is given by the new paradigm in reasoning (Over, 2009; also see Elqayam and Over, 2013, which is an introduction to a special issue in the new paradigm), which is based on Bayesian probability and dual processes. This new paradigm offers an alternative theoretical framework to those typically assumed in imaging research on deductive reasoning. In providing such a review, it is fortuitous that there has been a recent detailed meta-analysis of this area (Prado et al., 2011). I therefore concentrate on the findings of this meta-analysis, bringing in other relevant imaging results as they bear on the line of argument.

I first discuss why we might expect slippage between different levels of explanation in reasoning research in terms of Marr’s levels. Brain imaging is concerned with the implementational level whereas the new paradigm is a computational level theory. I then summarize the results of Prado et al.’s (2011) meta-analysis of 28 imaging studies. I then introduce the new paradigm and trace the consequences of its two critical features—(i) it is probabilistic and (ii) it invokes dual processes—for the interpretation of these brain imaging results. In doing so, I make several proposals. First, the main function of the core brain region identified by Prado et al. (2011) is most likely elaborative, defeasible reasoning not deductive reasoning. Second, the subtraction methodology and the meta-analytic approach may remove all traces of content specific System 1 processes thought by many to underpin much if not most human reasoning. Third, interpreting the function of brain regions activated by a task depends on our theories of the function that a task engages. When there are multiple interpretations of that function, interpreting what an active brain region is doing is not clear cut. Moreover, this issue is not resolvable at the implementational level. I conclude that imaging research may need to catch up with the computational level where there has been much recent progress.

## Computational levels

The multilevel nature of computational explanation in the cognitive sciences leads to multiple research strategies for investigating the cognitive processes that underlie any human behavior. At Marr’s (1982) computational level, the function that the mind/brain is believed to be computing in the performance of some task is specified. At the algorithmic level, the sequence of processing steps that compute this function is specified. At this level, various processing limitations need to be taken in to account, which may serve a critical explanatory role, e.g., working memory limitations. Finally, at the implementational level, the actual physical hardware in which the cognitive algorithm is instantiated in the brain is specified. At this level, the limitations of the physical components implementing the cognitive algorithm are taken into account, e.g., the time course of neural responses. As Marr envisaged these levels, addressing the computational level was the priority, i.e., the “function first” approach, because only this strategy was likely to prove successful. For example, little progress was made in understanding the operation of the heart until it was realized that its function was to circulate blood around the body. This multilevel nature of computational explanation means that researchers often pursue different research strategies that focus on only one level, usually determined by their own particular technical competences. This is usually unproblematic but it can create slippage between levels whereby research may proceed at different paces for a period of time, i.e., one level may move ahead while our understanding at the other levels lags behind (Chater et al., 2003).

In this paper, I argue that there has been slippage between the computational and implementational levels in the study human reasoning. Brain imaging research has largely appealed to theoretical frameworks at the computational level that over the last 20 years have been strongly challenged by the new probabilistic paradigm in human reasoning (Oaksford and Chater, 1994, 2001, 2007; Over, 2009; Elqayam and Over, 2013). In this paper, I examine what may be involved in re-aligning these levels of explanation in reasoning research.

## Imaging results: Prado et al.’s (2011) meta-analysis

In describing the existing research on the brain imaging of deductive reasoning, a good starting point is to briefly summarize Prado et al.’s (2011) meta-analysis. These studies initially presented a confusing set of results, which led (Goel, 2007, p. 440), to suggest that there may not be a unitary neural system for deductive reasoning, but rather “a fractionated system that is dynamically configured in response to certain task and environmental cues”. Prado et al.’s (2011) meta-analysis seems to reveal more consistency amongst these studies. They appear to show a core, mainly left lateralized, system being active in deductive reasoning with other subsystems being recruited dependent on the nature of the task, be it propositional, categorical, or relational reasoning. The core system involved the left lateralized inferior frontal gyrus (IFG), middle frontal gyrus (MFG), precentral gyrus (PG), posterior parietal cortex (PPC), and the basal ganglia (BG); it also included one medial structure, the medial frontal gyrus (MeFG). Prado et al. (2011) interpret this finding as consistent with the “left brain interpreter” hypothesis (Roser and Gazzaniga, 2006). The left hemisphere is primarily engaged in interpreting incoming information and filling in the missing information via inferential processes. The primary involvement of left lateralized brain systems seems to run counter to some accounts of human reasoning that place special emphasis on visual-spatial representations and processes, i.e., mental models (Johnson-Laird, 1983), which are primarily right lateralized.

Additional systems seem to be recruited for specific deductive tasks. Propositional reasoning involves relations between propositions like *if the key is turned, the car starts*, *the key is turned*, therefore, *the car starts*. This is the classical propositional inference of modus ponens and it depends purely on the connectives (*if…then* here but also *and*, *or*, *not*) and not on any deeper analysis of the propositions involved. Relational and categorical reasoning rely on going deeper in to the subject/predicate structure of a proposition. Categorical reasoning involves categorical statements like *All artists are beekeepers*, where “artists” is the subject and “beekeepers” is the predicate. This mode of reasoning is typically investigated using two premise quantified syllogisms such as *All artist are beekeepers*, *Some artists are smokers*, therefore, *Some beekeepers are smokers*. Relational reasoning moves from unary predicates, involving one variable, to relations, usually only binary, e.g., *John is taller than Fred*. These are typically investigated using the transitive inference paradigm—*John is taller than Fred*, *Fred is taller than Jane*, *is Jane taller than John*?—and spatial reasoning, e.g., *John is to the left of Fred*, *Fred is to the right of Jane*, *is Jane to the right of John*?

Relational arguments activate bilateral PPC and right MFG. Bilateral activation of the PPC is commonly seen in studies of visuospatial tasks and the reliable activation of right PPC in relational arguments seems consistent with theories like mental models. Categorical arguments only show strong activation of left lateralized IFG and BG and this activation is more consistent than for relational or propositional reasoning. These regions seem to be most consistently associated with processing syntax and grammar (e.g., Goel et al., 2000; Ullman, 2006; Grodzinsky and Santi, 2008). Propositional arguments are also left lateralized and most strongly activate PPC, PG, and MeFG. PPC and MeFG have been associated with non-syntactic verbal processing and maintaining abstract rules in memory respectively (Bunge et al., 2003; Booth et al., 2007).

Prado et al. (2011) draw an important conclusion from the finding that there is no one neural system apparently involved in all three domains of deductive reasoning investigated in these studies. No theory that suggests that these different domains all rely on a unitary underlying cognitive process is likely to be able to explain these results. Only some types of reasoning, apparently relational reasoning, seem to invoke visuospatial processing, propositional and categorical reasoning do not. They suggest that this tends to rule out unitary theories like mental logic (e.g., Rips, 1994) and mental models (Johnson-Laird, 1983) which propose that either formal rules or visuospatial representations underlie all deductive reasoning. Indeed, mental models theory makes the broader claim that such unitary visuospatial representations underlie all reasoning, deductive or inductive.

In most of the studies in Prado et al.’s (2011) meta-analysis, the theoretical rationale was to compare just two computational and implementational level theories of human reasoning. At the computational level, both mental models and mental logic theories take standard binary truth functional logic as defining the function the cognitive system is trying to compute.^1^ They diverge only on the nature of the representations and processes that implement this logic in the human mind i.e., they disagree primarily at the algorithmic level. Framing these investigations as deciding between these two theories also suggests that investigating deductive reasoning means to only study reasoning which can be captured by standard logic. However, it is arguable that over the last 15–20 years the most notable progress in the study of human reasoning has been at the computational level where alternative probabilistic theories of what people are doing in deductive reasoning tasks have been proposed (Hahn, 2014). These probabilistic accounts have become known as the “new paradigm” (Over, 2009; Manktelow, 2012). I now trace the origins of the new paradigm and its consequences for the interpretation of neuroimaging data.

## The new paradigm

There are two strands to the new paradigm. First, it is probabilistic. Second, it is a dual process theory that invokes both System 1 and System 2 processes (Evans, 2010; Stanovich, 2011). System 1 is Kahneman’s (2011) fast system and System 2 is his slow system. I look first at the probabilistic strand and its motivations and relate these directly to some of the results discussed in Prado et al. (2011).

### Probabilities

In motivating the probabilistic strand of the new paradigm, I begin with a quote from Dennett:

“But it is obviously true that most people never engage in explicit non-enthymematic formal reasoning” (Dennett, 1998, p. 289).

Enthymematic reasoning, for example, Tweety is a bird therefore Tweety flies, explicitly involves the use of world knowledge in order to fill in information not explicitly stated, i.e., that *all birds fly*, *normally birds fly* or *the probability that birds fly is high*. We make these inferences automatically with little conscious thought. As Dennett’s remark implies, this is the kind of inference that underpins our everyday lives and interactions with others. It also implies that the kind of “non-enthymematic formal” reasoning required in most of the reasoning tasks investigated in Prado et al. (2011) and in most deductive reasoning tasks used in the lab, are not commonly engaged in by the man or woman in the street. Consequently, attempting to derive a general theory of human reasoning by investigating these kinds of tasks is perhaps to step off on the wrong foot.

Concerns could be assuaged if this kind enthymematic reasoning could be captured by standard logic. However, one of the primary motivations for moving to probabilistic theories in the new paradigm has been the fact that enthymematic reasoning is defeasible (Oaksford and Chater, 1991, 2007). That is, learning that Tweety is an ostrich *defeats* the inference that Tweety can fly on learning that Tweety is a bird. We have rehearsed the problems of attempting to reconstruct such reasoning in standard logic many times before and do not do so again here (Oaksford and Chater, 1991, 1993, 1995, 2007). The probabilistic approach characterizes these inferences as being underpinned by probabilistic relations such as being a bird makes the probability that something flies high. That is, the world knowledge that underpins the enthymematic inference above is something like, *if x is a bird then x can fly*, where Pr(*if x is a bird then x can fly*) = Pr(*x can fly*|*x is a bird*) and this probability is high.

Another important aspect of this kind of reasoning, which Fodor (1983) calls non-demonstrative inference, is that it is the prototypical central cognitive process (Fodor, 1983; Oaksford and Chater, 1991). The contrast between modular and central cognitive processes is drawn along the lines of those that require large amounts of world knowledge and those that do not. Fodor (1983) argued that central cognitive processes are *Quinean*.^2^ A process is Quinean when it apparently invokes the whole of our belief system. So the reason we draw the inference that Tweety can fly is that this is the most *plausible* inference to draw. But plausibility is only definable against the backdrop of everything else we know or believe. Moreover, any Bayesian probabilistic account is going to be Quinean. Our best bet about how we determine someone’s subjective probability Pr(*x can fly*|*x is a bird*) is given by the Ramsey test. This test involves assuming Tweety is a bird, i.e., adding this proposition to our stock of beliefs while making minimum adjustments to our other beliefs, and reading off our new degree of belief that Tweety flies. This is a philosophical prescription but its implications for psychological processes are clear: defeasible reasoning, probabilistically construed or not, must invoke central cognitive processes.

#### Imaging, inference and central cognitive processes

This brief account of the underlying motivations for the probabilistic strand of the new paradigm (see also, Oaksford and Chater, 2007, Chapters 1–4) leads to two conclusions that appear to be supported by the imaging results discussed by Prado et al. (2011). First, Prado et al. (2011) identify their left lateralized core system with Gazzaniga’s “left brain interpreter” hypothesis (Roser and Gazzaniga, 2006). *It is important to be clear on the nature of the inferences that underpin this hypothesis*. A main source of evidence for the left brain interpreter hypothesis is the *elaborative* inferences that some patients and normal participants make in interpreting pictures. These elaborative inferences seem to be responsible for false recognition of novel pictures as being previously viewed. Of course, our enthymematic inference that Tweety can fly is an elaborative inference of precisely this sort. It could only be construed deductively if the enthymematically provided premise was ***all**** birds can fly* but then it would not be defeasible. But all elaborative and enthymematic inferences are defeasible and people may not even be aware of the fact that they have drawn one until it is overturned, e.g., on being told Tweety is an ostrich, and the mild sense of surprise that they then experience. In sum, if the left brain interpreter hypothesis is correct as an interpretation of the brain imaging results, then its primary function is probably not in deductive reasoning but rather elaborative, defeasible, and probabilistic reasoning. At least this is the kind of reasoning that has provided the principal evidence for the left brain interpreter hypothesis in the past.

Second, such defeasible, probabilistic reasoning, as we have just discussed, is perhaps our best candidate for a central cognitive process. That is, it is one of the processes that is least likely to be subserved by a unitary cognitive module. And this would appear to be exactly what the brain imaging data reveals, reasoning is not subserved by a unitary cognitive process, be it formal rules or visual spatial representations, in a single isolable module. It is also worth noting that, given the defeasible, probabilistic nature of the inferences that underpin the left brain interpreter hypothesis, when deployed in deductive tasks this brain system is probably not being used to perform functions for which it originally evolved. That is, at best, deductive reasoning is a limiting case of this system’s primary function, for example, when the probabilities go to 0 or 1.

#### Deductive tasks

A possible objection to the line of argument in the last section is that the imaging results reviewed in Prado et al. (2011) specifically focused on deduction, i.e., the tasks were very specifically deductive tasks, which could not form the evidential basis for generalizing to defeasible non-demonstrative reasoning. However, in the reasoning literature mental models theory *has* taken these tasks to provide the basis for a wholly general theory of reasoning subsuming deduction (Johnson-Laird and Byrne, 1991), probabilistic inductive reasoning (Johnson-Laird et al., 1999), causal reasoning (Goldvarg and Johnson-Laird, 2001) and much else besides. Moreover, mental logic and mental models are the theoretical frameworks on which the imaging research has primarily concentrated. The new paradigm argues that because everyday, defeasible reasoning is the ubiquitous phenomena people apply sensible reasoning strategies for dealing with the everyday world to laboratory deductive reasoning tasks. This strategy can explain away many of the so called biases observed in human deductive reasoning (Oaksford and Chater, 2007).

Could it nonetheless be argued that the specific tasks used in the imaging studies review by Prado et al. (2011) are uniquely deductive and consequently they genuinely investigate just this very narrow domain of human reasoning? A point I elaborate on further below, is that we require a computational level theory to define the function that a task engages (*Functions, Tasks, and Active Regions*). In imaging research, “deduction” is taken to refer to binary truth functional logic as it is in mental logic and mental model theory. But there are a range of alternative logics especially for the conditional (see, e.g., Haack, 1975; Bennett, 2003) and there are well specified probabilistic accounts of categorical reasoning (Chater and Oaksford, 1999). Moreover, there are varieties of probability logic (Adams, 1998) in which coherent probability intervals are *deduced* from probability assignments to the premises (Pfeifer and Kleiter, 2010; Pfeifer, 2013). Such logics are just as deductive as binary truth functional logic.

Perhaps it could be argued that at least tasks like relational and spatial reasoning have deterministic binary logical solutions and as such are genuinely “deductive” tasks in the sense intended in mental logic and mental models theory. However, phenomena like perspectival relativity (Barwise and Perry, 1983) question this view. Take, for example, the premises *John is to the left of Fred*, *Fred is to the right of Jane* which is assumed to lead to the deterministic logical conclusion that *Jane is to the right of John*. If *Jane* and* John* are both facing each other with Fred in the middle facing neither then the question of whether *Jane is to the right of John* has no deterministic answer, they are neither to the left nor to the right of each other, despite the truth of the premises. Left and right depend on our subjective frame of reference in personal space. Another example is if *Fred* is standing at the North pole and *Jane* and *John* at the South pole. In this case, *Jane* and *John* would appear to be simultaneously to *Fred’s* left and to his right. Such counterexamples suggest that there are certain orientations that make the conclusion more likely but it does not follow deterministically. Even relations like *taller*, which rely on being able to measure the world, may require a probabilistic theory. Measurement error suggests that our representations of items on a scale use distributions which may overlap. Such representations can explain the symbolic distance effect where for a long transitive chain, e.g., *a > b > c > d > e* (“>” = is taller than), people find it harder to discriminate whether *c > d* than *a > e* (Cohen Kadosh et al., 2005). In summary, tasks are not deductive in and of themselves. What function a task engages is determined by the empirically most adequate computational level theory of that task.

#### Imaging: deduction vs. induction

We have argued that the core system identified by Prado et al. (2011) is concerned with defeasible, non-demonstrative reasoning. The new paradigm has been characterized as “imperialistic” (Rips, 2002) in that it attempts to assimilate deduction to probabilistic inductive reasoning. However, there is behavioral data suggesting that these processes dissociate (Rips, 2001; Heit and Rotello, 2010). Although recently Lassiter and Goodman (2015) have shown these differences may have more to do with the semantics of the terms used to elicit people’s responses, i.e., is the conclusion “necessary” (deduction) or “plausible” (induction), than with fundamental differences in the reasoning process which remains probabilistic. A suggestion originally made by Oaksford and Hahn (2007). There is also imaging data relevant to this question.

Goel and Dolan (2004) found that some structures were more active in deduction (left IFG) than in induction and that some were more active in induction (primarily left MFG) than in deduction. They argue that their findings are more consistent with other studies, particularly lesion studies, than previous work apparently showing that these modes of reasoning were lateralized with induction associated with the left hemisphere and deduction with the right (Parsons and Osherson, 2001). Goel and Dolan’s (2004) studies were included in Prado et al.’s (2011) meta-analysis and both these structures are part of the core system they identified. Goel and Dolan argue that left IFG is associated with Broca’s area and hence language, working memory and perhaps syntactic processing. Left MFG activation, they hypothesize is associated with the recruitment of general knowledge required for induction.

Induction and deduction activate much the same brain system. Moreover, given the nature of these inferences even what differential patterns of activation there were are understandable. The new paradigm does not deny that deduction and induction are distinct (Evans and Over, 2013). Deduction involves inferences over the syncategorematic or logical terms of a language (*if…then*, *and*, *or*, *not*, *all* etc.), i.e., the inference follows from the meaning of these terms. This is not the case for the inductive inferences that Rips (2001), Goel and Dolan (2004), and Heit and Rotello (2010) investigated which involved categorical induction. In deduction processing of the structure of premises is important but it is less so for the premises of an inductive inference which may simply present a string of facts (e.g., domestic cats have 32 teeth, lions have 32 teeth). Moreover, we learn about the world by observation in a similar way, i.e., inductive inferences do not have to be mediated by language in the way deductive inferences are. In probability logic, the meaning of the conditional is given by the conditional probability. The assertion of a conditional means that that the conditional probability is high. So while both inferences types are probabilistic, and both rely to a degree on world knowledge, there is an important structural difference between induction and deduction, which is what Goel and Dolan’s result are presumably picking up. A final observation is that we can find no lesion study showing a full double dissociation between induction and deduction. Although Goel and Dolan cite one case study involving a single dissociation using a theory of mind task (Varley and Siegal, 2000), no classical deductive or inductive reasoning tasks were used.

### Dual processes

In the new paradigm, it is agreed that a dual process theory is required (Evans and Over, 2004; Evans, 2010; Oaksford and Chater, 2010, 2011; Stanovich, 2011). System 1 is implicit, probabilistic, and based on world knowledge. System 2 is explicit, involves working memory, and is based on “analytic” processes. These analytic processes have been argued to be either also probabilistic (Evans and Over, 2004; Oaksford and Chater, 2009, 2010, 2011; Evans, 2010; Pfeifer and Kleiter, 2010) or based on standard binary logic (Rips, 1994, 2001; Stanovich and West, 2000; Heit and Rotello, 2010; Klauer et al., 2010; Stanovich, 2011). Whatever view one takes, it is generally agreed that deductive reasoning behavior is a product of an interaction between both these systems.

Kahneman (2011) uses some instructive examples to illustrate the nature of System 1 and System 2 processes. To illustrate System 1, he simply presents the juxtaposition of two words:

Banana Vomit

As he observes, a whole panoply of responses are triggered automatically by this juxtaposition. A whole causal story is probably constructed connecting the ingestion of bananas and vomiting. Moreover, a mild sense of surprise is invoked by this unusual juxtaposition. Unpleasant visual and auditory images will also be briefly triggered. The processes that produce these reactions happen unconsciously and very rapidly, all we are aware of is a reaction. He illustrates System 2, by tasks like counting back in threes from say 1037. This task is effortful, fully conscious, difficult to keep going, and involves applying the rules of arithmetic. Tasks illustrating the interaction of these systems are those like the bat and ball problem. In this task participants are told that the bat costs a dollar more than the ball and that together they cost $1.10 and they are asked how much does the ball cost? A spontaneous System 1 response is ten cents, which must be wrong because this would make the total cost of the bat and ball $1.20. In such tasks, the automatic System 1 response may need to be overridden and the actual cost consciously calculated in System 2.

In deductive reasoning tasks, it may be that a spontaneous System 1 response needs to be overridden but it seems unlikely that lay participants are capable of then engaging the correct logical rules in System 2 as they can the rules arithmetic for the bat and ball problem. Except for the logically trained these rules are simply not consciously available (of course for the bat and ball problem to be solvable, the rules of arithmetic also had to be learned). Consequently in deductive reasoning performance, it is probably best not to consider System 2 as conscious. This seems consistent with recent work on *logical intuitions* which shows that people appear to unconsciously detect the conflict between the intuitive System 1 response and the correct response even if they make the apparently biased System 1 response (De Neys, 2012, 2014). What people will be conscious of is a response, initially triggered by System 1, accompanied by a *feeling of rightness* (Thompson et al., 2011). This feeling may well depend on how the intuitive System 1 response agrees or conflicts with the output of System 2.

A great deal of work in the new paradigm is on showing that apparently irrational performance on many tasks is actually rational from a probabilistic perspective. Moreover, much of this behavior is hypothesized to be the responsibility of System 1. Kahneman’s illustrative example of System 1 in action suggests that much of the information required by a rational theory of inference and decision is automatically computed at this level. For example, to understand the juxtaposition of just these two words people seem to generate a causal model relating the ingestion of bananas to vomiting. Moreover, a surprising event is one that is improbable, which suggests that relevant probabilities are automatically computed. Furthermore, people have a spontaneous emotional reaction to this juxtaposition expressing relevant hedonic or experienced utilities. The almost immediate availability of all this information may suggest that System 1 is indeed capable of some complex inferential processes, consistent with logical intuitions (De Neys, 2012, 2014).

Recently, it has been suggested that System 1 uses this information in inference in a similar way to the unconscious inferences involved in perception and action hypothesized by Helmholtz (Oaksford, 2014, Submitted). Again most progress on unconscious inference is being made at the computational level by computational biologists. These unconscious inferential processes are being understood in probabilistic terms in the Bayesian brain hypothesis (Dayan and Hinton, 1996; Friston, 2005, 2008; Clark, 2013). In brief, perception is viewed as the process of using alternative generative models of the current context to generate hypotheses about the causes of the pertubations of our sensory surfaces. These hypotheses, e.g., it is a dog or it is a cat, are at the top level of a hierarchical Bayesian model and these cascade down making lower level predictions ultimately for the responses of center surround units in our sensory receptors. Prediction errors, e.g., the hypothesis says the unit should be on when it is off, are then fed back up the hierarchy minimizing expected surprise or entropy concerning the cause of the proximal stimulus, i.e., the least surprising interpretation is adopted. It has also been shown how these cascaded inferential processes can be implemented in cortex.

In sum, most reasoning is largely unconscious, it occurs automatically based on the rich information generated by System 1 which also seems directly implicated in unconscious inferences in perception and action. Our theories of System 1 in the psychology of explicit verbal reasoning and our theories of unconscious inference in perception and action also converge on a Bayesian account.^3^ This means that content, which fixes the relevant probabilities, is central to the reasoning process. But most imaging studies have framed their investigations in term of mental logic and mental models in which content is largely irrelevant. As I now argue, this fact may have important consequences for the interpretation of imaging results in the psychology of verbal reasoning.

#### Imaging system 1

Most brain imaging studies use the subtraction methodology to isolate brain regions that are specific to deduction and this usually involves contrasting materials with relevant content. So for example, in Goel and Dolan (2003) experiments on belief bias in categorical reasoning, materials like:

(A) No reptiles can grow hair
  Some elephants can grow hair

So, No elephants are reptiles (true conclusion, invalid inference) were contrasted with a baseline:

(B) No reptiles can grow hair
  Some elephants can grow hair
  No fried foods have cholesterol

Subtracting out activation due to this baseline may remove any traces of the automatically activated content based processes like those involved in Kahneman’s System 1 example. These processes are automatically activated by the content of the words which are also present in the contrast. But if most of the inferential action is at the System 1 level this means that the subtraction methodology may be removing most activations of interest (see also, Monti and Osherson, 2012, for a similar line of argument). Other contrasts that have been used, e.g., a simple fixation location, may seem to avoid this problem. However, even if such contrasts retained activations associated with content, the goal of Prado et al.’s (2011) meta-analysis was to detect active regions *across* studies. Consequently, these content based activations will be removed in the meta-analysis because content varied between studies (and indeed between tasks).

Content-based System 1 activations may be subject to a great deal of variation not only across studies but also across individuals. Would one expect, for example, there to be much spatial overlap between two people’s representations of the concept “horse”? When one thinks of horses, regions associated with their shapes, movements, smells, and locations where they have been encountered are activated and binding these disparate responses together is the crucial step in having the concept “horse”. Given what is likely to be a diffuse pattern of activation, presumably involving different sensory centers and memories, it seems unlikely that there will be much spatial overlap in regions activated across individuals, especially given the good spatial resolution of fMRI. Presumably this information is lost as a result of aggregating across individuals: even though each individual is doing the same thing slightly different brain regions are active.

Some studies support this contention. Having people think of a particular concept, e.g., “horse,” leads to diffuse activation of many regions across the whole brain (Pereira et al., 2011). Pereira et al. (2011) also showed that at a certain level of abstraction these activation patterns could predict the topic being thought about and words associated with those topics. This was achieved by extracting a latent topic model from Wikipedia articles. Using machine learning technique a mapping was learnt between the latent factors that summarized the articles and patterns of distributed brain activity. This mapping could then be inverted to use the pattern of brain activity to predict the topic being thought about and hence words associated with that topic. Consequently, at quite a high level of abstraction there may be some consistency between topics being thought about and the spatial distribution of activation in the brain. However, we know of no work that relates individual concepts, such as “horse” to consistent patterns of activation across individuals. Moreover, the simple fact that these activations do not survive the subtraction methodology used in the reasoning studies summarized by Prado et al. (2011) suggests that across individuals there is little consistency in the brain regions activated.

The notion that for many different concepts and events people’s own unique experience may fail to lead to patterns of brain activity that generalize fully across individuals is consistent with the subjective nature of probabilities in the new Bayesian paradigm. Our own unique experiences mean we may assign quite different probabilities to the same events. Indeed, if we did not differ in our beliefs in this way then there would be nothing to argue about at the social level where, it has been argued, most reasoning goes on (Hahn and Oaksford, 2007; Mercier and Sperber, 2011).

In summary, these imaging studies are not recording System 1 in action.

#### Functions, tasks, and active regions

I have concentrated so far on what imaging studies may miss in investigating System 1 processes. Before moving on to look at the difficulties in interpreting the activations that remain, I pause briefly to consider the relationship between cognitive tasks, the functions they engage and the interpretation of active brain regions. I argue that (i) function comes first, and two (ii) the function a task engages may be in dispute. In the next section, I trace the consequences of (i) and (ii) for the interpretations of the regions identified by Prado et al. (2011).

Function is assigned partly historically. For example, in investigating belief bias, Goel and Dolan (2003) contrasted correct and incorrect performance on trials that show a conflict between the validity of an inference and the truth of the conclusion (see (A) and (B)). One contrast revealed activation of right inferior prefrontal cortex (rIPFC) and the other of ventromedial prefrontal cortex (VMPFC). How do we interpret such findings? This question is answered partly in terms of the nature of the current task but also in terms of past history. So rIPFC is active when correct responses are made to conflict problems implicating inhibitory processes consistent with previous results. VMPFC is active when incorrect responses are made to conflict problems implicating intuitive, emotional processes, again consistent with previous results. The functions assigned to these regions are partly based on computational level assumptions. These determine the “correct” response and the assumption that “inhibition” is required to identify the correct response. But it is also based on history, what tasks (with assumed functions) have activated the region in the past. While this is all perfectly reasonable, there are potential problems.

First, there is the problem of a general historical bias. Just because a certain type of task, *t_1_*, with a certain presumed function, *f*_1_, was first found to activate a region, *r*_1_, then this is the function associated with that region. But this is simply a historical artifact. If the current task, *t*_2_, with presumed function, *f*_2_, had been investigated first and found to activate region *r*_1_ then *f*_2_ would be the function presumed to be engaged when this region is activated and *t*_1_ may be assumed to engage *f*_2_ as well as *f*_1_.

Second, this line of argument suggests that interpreting imaging results requires us to be very clear on the functions that cognitive tasks engage. Moreover, if this is clear then function drives interpretation. If region *r_1_* is activated by *t*_2_, even though it has been previously associated with *f*_1_, it must now be regarded as also computing *f*_2_. At least there is no reason, other than history, to argue that instead *t*_2_ engages *f*_1_. Moreover, in cognitive science, and in particular deductive reasoning, the task/function relationship may be in dispute. So called deductive tasks, say *t*_1_, are being interpreted as not engaging deduction, *f*_1_, but rather probabilistic reasoning, *f*_2_. We can only interpret the function of a brain region in terms of the tasks that engage those functions and activate that region. If our theory of the function engaged by a task changes, then so does our interpretation of what active brain regions are doing. For example, later on I argue that the computational level assumptions underlying the interpretation of belief bias results (Goel and Dolan, 2003) may be wrong (*NIRS, TMS and Belief Bias*). Imaging studies are only informative against the backdrop of a computational level theory of the tasks used in these studies. Consequently, whatever one’s preferred research strategy, i.e., whether you concentrate on the implementational, algorithmic, or computational level, function comes first.^4^

#### Imaging beyond system 1

Against the backdrop of these last two arguments, I now consider the other patterns of activation that Prado et al. (2011) found with relational, categorical and propositional reasoning. With relational arguments, in particular in transitive inference, e.g., A is taller than B, B is taller than C…etc, is C taller than A?, Prado et al. (2011) found activation of bilateral PPC and right MFG consistent with the use of visual representations. Although this finding has recently been qualified by results showing that when the transitive chain involves quantifiers, all A are B, all B are C…etc, only left hemisphere activation is found (Prado et al., 2013). These findings suggest, what many researchers have suspected, that relational and spatial reasoning are not part of our core reasoning system. Rather when such arguments can be easily represented visually the mind/brain exploits this fact but this is a specific strategy. Moreover, as Prado et al. (2013) have shown, when this strategy is difficult, i.e., when the transitive chain involves whole sets and not individuals, the system reverts to the left brain interpreter.

Prado et al.’s (2013) results also argue against the mental model theory of quantified syllogistic reasoning. In this account, categorical reasoning proceeds over an imagistic representation of a small number of arbitrary exemplars of the sets described by the quantifiers. So according to mental model theory both categorical reasoning and relational reasoning should engage right lateralized systems. In contrast, the main probabilistic account of categorical reasoning, the probability heuristics model (Chater and Oaksford, 1999; Oaksford et al., 2002), suggests that a simple set of probabilistically motivated heuristics operate over linguistic representations of the premise and conclusion. Prado et al.’s (2011) results for categorical reasoning are consistent with this account. They show strong activation of left lateralized IFG and BG, regions most consistently associated with processing syntax and grammar. The heuristics in PHM select a syntactic conclusion frame using probabilistically motivated heuristics and then use other heuristics to determine the order of end terms in this syntactic frame (Oaksford et al., 2002). These heuristics depend on an ordering over the informativeness (the inverse of probability) of the premises. Specific content has the potential to alter this informativeness ordering leading the heuristics to make different predictions. While this possibility has never been experimentally tested, it shows that even this relatively abstractly defined probabilistic theory still relies on System 1, i.e., on content.

Prado et al. (2011) found that propositional arguments most strongly activate PPC and MeFG which have been associated with non-syntactic verbal processing and maintaining abstract rules in memory respectively. Perhaps the most researched and important area in propositional reasoning is conditional reasoning, i.e., reasoning using what is rendered in English as *if…then*. Most recent research has involved causal conditional reasoning, where it is clear that the specific contents are important. However, conditional reasoning has also been extensively researched using abstract materials, which seemingly could not engage content. The fact that regions associated with maintaining abstract rules in memory are activated suggests that perhaps formal syntactic processes are directly involved. There are good arguments against this interpretation.

First, as I have argued, the functions engaged by a brain region may well be in dispute. Whether we need to use abstract rules in language processing or reasoning is contentious. In language processing the debate has raged since the advent of neural networks in the 1980s (Rumelhart, 1986). The issues hinge on whether generalization is achieved by abstract general rules or by similarity and analogy to pre-existing knowledge. Thus, as we discussed above, whether MeFG co-ordinates the processes involved in computing similarity and analogy or storing abstract rules is contentious from this perspective. An interesting prediction is that if computing similarity and analogy is involved in reasoning with abstract material one might expect more rather than less general knowledge to be activated. As materials become more abstract they will be similar to more of what we know, e.g., to all domains we tend to describe using conditionals. We may find an answer to this question once appropriate methods to image System 1in action are used.

Second, it seems doubtful that humans have evolved a specific module for handling abstract logical rules of inference that are the product of the last two millennia of logico-philosophical labor. Formal logic is a cultural product, a tool, for reasoning with pencil paper or computer. It is not the workings of the human mind made concrete in symbols. The *if…then* construction is used ubiquitously because it can be used to describe the various relationships or dependencies in the world, like causes, dispositions, intentions, regulations and so on, which allow us to predict what will happen next and to explain why what happened happened. The reasoning mind is likely to be very concrete constructing specific small scale models of reality in System 1, like Kahnemen’s banana-vomit example or using specific relations, and reasoning over these (Oaksford and Chater, 2013, 2014; Oaksford, 2014, Submitted). These last two points make the argument that there are functions, *f*_2_ and *f*_3_, that are in contention to account for the tasks that engage MeFG. Consequently, there is reasonable doubt about whether it engages abstract rules.

I finish this section by looking again at the function of the core brain system identified by Prado et al. (2011). As I argued above, it seems unlikely that either System 1 or System 2 processes in most “deductive” reasoning tasks are like consciously performing mental arithmetic like that required to solve the bat and ball problem. However, in all reasoning tasks the results of these processes must become conscious and be turned into a verbal response to be delivered verbally (production task) or to match to a range of possible response options (selection task). What becomes conscious may also be a feeling of wrongness when the outputs of System 1 and System 2 conflict.^5^ This would seem to be the shared common core of most reasoning tasks. But of course it is the final stage not the actual core of the reasoning process.

### Further imaging studies

So far I have only discussed the fMRI localization studies included in Prado et al.’s (2011) meta-analysis. However, there are other imaging studies using fMRI and other imaging techniques, such as EEG using ERPs, Infra-red Spectroscopy (NIRS) and Transcranial Magnetic Stimulation (TMS), which are relevant to the dual-process aspect of the new paradigm. In this section, I deal with these further studies by the imaging technique used and then by the task/functions investigated.

#### fMRI studies

Here I look at further fMRI studies used to investigate (i) component process of deductive reasoning and (ii) the matching effect (Evans and Lynch, 1973; Oaksford and Stenning, 1992).

##### Component processes

Some fMRI (Fangmeier et al., 2006) and lesion studies (e.g., Reverberi et al., 2009) have concentrated on the component processes of deductive reasoning. Reverberi et al.’s (2009) lesion study was broadly consistent with the conclusion of Prado et al. (2011) that the right hemisphere and imagistic processing are not part of the core reasoning system. Right frontal lesions did not impair deductive reasoning. Patients with left frontal regions and impaired working memory did show deficits. More revealing evidence distinguishing the fast System 1 from the slow System 2 would be expected from studies investigating the time course of reasoning. Fangmeier et al. (2006) investigated the component processes of deductive reasoning separating out premise presentation, premise integration, and validation. These stages were defined by the timing of the presentation of two premises in visually presented spatial linear syllogisms, e.g., premises: V X (after 2 s), X W (after 6 s), conclusion: V W? (after 10 s). Perhaps unsurprisingly, given the visual presentation of premises, the premise presentation phase activated left and right occipital lobes. Premise integration and validation phases shifted activation toward frontal structures. As I have remarked, these purely visuospatial tasks are unlikely to invoke the same reasoning processes that underlie human *verbal*, reasoning. Moreover, the lack of content and the artificial pacing of the stimulus presentation to allow data collection using the relatively poor temporal resolution of fMRI are unlikely to be very revealing of the rapid System 1 in action.

##### Matching effects

There have been studies looking at phenomena that have provided evidence for dual processes, in particular, the matching effect (Evans and Lynch, 1973). Matching occurs when negations are included in the sentences used in a reasoning task. Usually these are in conditionals, e.g., *if there is an H then there is not a circle*. If asked to construct a falsifying instance of this rule people find it relatively easy because the falsifying instance, H and circle (a True/False instance TF), perceptually matches the named items in the rule. However, if they are asked the same question with the rule, *if there is an H then there is a circle*, then they find it more difficult. The TF instance is, e.g., H and square (or any non-circle), which does not completely match the named items. In a PET study, Houdé et al. (2000) showed that prior to perceptual inhibition training, this task primarily activated occipital visual regions, consistent with perceptual matching, but post inhibition training activation shifted to more frontal areas. More recently, Prado and Noveck (2006, 2007) have used fMRI to investigate the matching phenomenon. Prado and Noveck (2007) used a novel parametric variation approach identifying brain regions whose activation varied with the number of mismatches or negations in a rule. They also showed that frontal regions, which became more active with more mismatches, showed decreases in their interactions with visual cortex, consistent with inhibiting matching. Perceptual matching can be regarded as one of the perhaps many subsystems of System 1 (Stanovich, 2011) and the frontal systems that inhibit this system is System 2.

The new paradigm is an evolving body of theory and there is active disagreement over the interpretations of some phenomena. Evans (2003) cites Houdé et al. (2000) as support for the dual process theory. However, there are many reasons to doubt that these PET and fMRI studies are recording System 1 in action. First, there is a very close overlap in the regions activated in Houdé et al. (2000) pre-intervention phase and in Fangmeier et al.’s (2006) premise presentation phase. Of course, it is not surprising that presenting premises activates visual areas as written language is still a visual stimulus. There is no immediate reason to think that activity in these regions should be a source of reasoning bias. Second, matching is a far more nuanced phenomenon than described in Houdé et al. (2000) and in Prado and Noveck (2006, 2007). For example, in the original studies (Evans, 1972; Oaksford and Stenning, 1992) it occurs only for falsifying trials, like the example in the last paragraph. However, verifying trials (constructing True/True instances) show a similar pattern of mismatches as for falsifying trials. So, if the matching phenomenon were a simple perceptual matching effect then both types of trial should reveal the bias. Third, much simpler manipulations than inhibition training remove this bias. For example, using real world thematic content rather than abstract alphanumeric stimuli or shapes removes the bias (Oaksford and Stenning, 1992). This simple fact suggests that matching is not a major factor in biasing everyday reasoning. Moreover, making it easier to identify the “contrast class” for a negated constituent removes the bias. Logically the contrast class for *there is not a circle* can be anything, literally, that is not a circle (e.g., a coal scuttle). But in context it is clear that another shape is intended. If there were only two shapes and participants knew this, then matching is likely to disappear, as it does when using rules like *if there is a vowel, then there is not an even number* (Oaksford and Stenning, 1992). A number that is not even is obviously odd. Prado and Noveck (2007) did detect areas that were differentially active depending on the number of negations, i.e., right anterior pre-frontal cortex, and suggest that this may be involved in computing contrast classes.

Oaksford and Stenning (1992), (see also Oaksford and Moussakowski, 2004) argued that the matching phenomenon is part of the normal process of computing contrast classes which is made difficult by the use of abstract material. They also show how this account combines with the probabilistic component of the new paradigm to explain matching effects both in the Wason selection task (Oaksford and Chater, 1994, 2003, 2007) and in the conditional inference task (Oaksford et al., 2000). Constructing contrast classes is part of the System 1 processes involved in generating probabilities.

Why do these imaging studies show the effects they do, i.e., mismatches correlated with regions that are inhibiting visual areas? I suspect that this is part of the much more general phenomenon of suppressing distracting information in attentional control. If shown a picture of a white bear (Wegner, 1994) and told not to think about it, all you can think about is white bears. Similar patterns of activation are likely to occur on many tasks requiring the suppression of distractors regardless of whether they are reasoning tasks. Moreover, suppression in the visual modality can be made more difficult in the presence of noise in the auditory modality. There is also work on the neural basis of these effects (Smucny et al., 2013) which reveals similar interactions between brain regions as shown by Prado and Noveck (2007). fMRI scanners are very noisy places and PET scanners are also quite noisy. Consequently, while being scanned these attentional effects would be expected to be even more pronounced and to dominate the normal processes of contrast class construction. In normal discourse, a whole range of phonetic, syntactic, semantic and pragmatic factors contribute to making contrast class construction easy (Oaksford and Stenning, 1992). It is only in abstract tasks where these supports are removed that matching is observed.

In sum, there is good reason to doubt that these studies of matching bias tap into the fast System 1 responsible for the effects in Kahneman (2011) anecdotal example and in contrast class construction (although Prado and Noveck (2007) show some evidence for the localization of these latter processes). Rather the primary effects observed seem to be concerned with the general suppression of distractors observed in many tasks which are exacerbated by the noisy environment of the scanner.

#### NIRS, TMS and belief bias

The studies we looked at in the last section all used fMRI which has limited temporal resolution and so is perhaps unlikely to reveal much about fast System 1 processes. Where they have been revealing on System 2 processes this has primarily involved the function of dorsolateral pre-frontal cortex in inhibiting distracting information emanating from visual areas not of the analytic processes thought to require working memory. Perhaps a better insight into the neural processes involved at the interface between System 1 and System 2 might be found using imaging methods with greater temporal resolution. In this section, I briefly look and working using near infra-red spectroscopy and TMS.

A series of four studies using NIRS by Tsujii et al. investigated the role of inferior frontal cortex (IFC, which includes the IFG) in the belief bias effect (Tsujii and Watanabe, 2009, 2010; Tsujii et al., 2010, 2011). This effect has also been assumed to provide evidence for dual processes. The effect is usually investigated using quantified syllogisms which can be systematically varied along the binary dimensions of validity (valid, invalid) and believability of the conclusion (believable, unbelievable). For example, *No mammals are birds*, *All dogs are birds*, therefore*, No dogs are birds* is valid and believable, whereas *No pigeons are mammals*, *All pigeons are birds*, therefore*, No birds are mammals* is invalid and believable. The belief bias effect is an interaction effect (Evans et al., 1983) such that people endorse invalid believable conclusions as much as valid believable conclusions (92% in both cases), whereas they endorse valid unbelievable conclusions (46%) far more than invalid unbelievable conclusions (8%). Accuracy is far greater for congruent trials (valid/believable and invalid/unbelievable, 92%) than for incongruent trials (valid/unbelievable and invalid/believable, 37%). In these imaging studies accuracy on congruent and incongruent trials was the behaviorial dependent variable. Incongruent trials require the System 1 belief based response to be inhibited to allow the System 2 analytic response to be made.

In Tsujii et al.’ studies they used manipulations to impair working memory performance either by using a dual task (Tsujii and Watanabe, 2009), time restrictions (Tsujii and Watanabe, 2010), or by using repetitive TMS on the IFC region (Tsujii et al., 2010) thought to be involved in working memory. High dual task load, short time restriction, and right IFC rTMS stimulation led to less accurate performance but only on incongruent trials. High dual task load and a short time restriction also reduced IFC/IFG activation but only in the right hemisphere. These findings suggest that right IFG is required to inhibit the System 1 heuristic or belief based response. In a further study, Tsujii et al. (2011) also used rTMS on the superior parietal lobule (SPL) as well as IFG using the belief bias paradigm. Stimulation in this region impaired performance on abstract syllogisms and incongruent trials, which they suggest require analytic System 2 processes. Tsujii et al. conclude that the function of right IFG is in inhibiting belief biased responding, the function of left IFG is a language area responsible for semantic processing and belief bias, while the function of bilateral SPL is analytic reasoning.

There are several points to make about these NIRS studies. First, the activations were integrated over a period lasting over a minute and so are not looking at rapid processes of the type that underlie Kahneman’s System 1. Second, the results are not consistent with previous fMRI studies. For example, the seat of inhibitory processing has moved from DLPFC (BA 46) in Prado and Noveck (2007) to right IFG (BA 44, 45, 47). Moreover, there seems to be little evidence of Prado et al.’s (2011) core left lateralized deductive reasoning system. Further problems of interpretation arise from the interactional nature of the belief bias phenomenon.

Recently, Dube et al. (2010) showed that the belief bias interaction has been misinterpreted. They show that the interaction effect observed in belief bias is consistent with curvilinear ROC curves. Properly analyzed, accuracy remains the same between conditions, and believability effects are pure response biases. They argue that their modeling results, “provide support for processing theories of deduction that assume responses are driven by a graded argument-strength variable, such as the probability heuristic model proposed by Chater and Oaksford (1999).” Their results are also consistent with probabilistic single function dual process theory (Oaksford and Chater, 2012, 2014). There is a clear distinction between processes based on long term memory for our beliefs about the world and processes that require working memory. However, the single function approach argues that these processes, where they concern reasoning, are both probabilistic.

Dube et al.’s (2010) analysis shows that the belief bias phenomenon that underpins the theoretical framework (logical analytic System 2 and belief based/heuristic System 1) used to interpret Tsujii et al. results, may not actually exist. A similar state of affairs exists in the study of optimism bias (Weinstein and Klein, 1996) where proper statistical analysis (Harris and Hahn, 2011) has shown that this phenomenon, apparently investigated in many imaging studies (e.g., Sharot et al., 2011), may not actually exist. These re-analyses of these phenomena are at the computational level, i.e., they show that the actual functions being computed in these tasks may not be what they first seemed. As we argued in the section *Imaging beyond System 1*, theories of function drive the interpretation of these imaging results, i.e., its function first. Consequently the interpretation of Tsujii et al. results may need to be re-thought.

A paper aimed at making general theoretical points about the current state of imaging research into deductive reasoning is not the place to offer such a re-interpretation of these results. However, it is worth observing that the interpretation is going to be further complicated by the fact the that people seem to unconsciously process both the nominally analytic and heuristic responses as evidenced by the activation of brain regions associated with conflict detection, i.e., the anterior cingulate cortex, whether people make the supposedly biased response or not (De Neys et al., 2008). That is, both possible responses seem to be computed in System 1. Such findings tend to suggest that System 2 doesn’t so much do analytic reasoning as adjudicate between possibilities and form a response (Oaksford, 2014, Submitted).

In sum, a major problem for imaging research is that there seem to be no onus to explore all the possible computational level interpretations of any set of results. Moreover, there is only a very loose connection between function and the activity of brain regions assumed to compute it. For example, the inference to SPL being the seat of analytic reasoning is based on a statistical tendency for rTMS stimulation of that region to impair abstract and incongruent tasks. In the light of Dube et al.’s analysis, it is very difficult to know what to make of this result. However, it most certainly does not tie this region to making deductive inference in a mental logic.

#### ERP and conditional inference

To explore the brain systems involved in the rapid System 1 processes, event related potentials recorded using EEG would seem to be the most promising route. The temporal resolution is excellent and many of the evoked waveforms have a well understood interpretation developed over many years of research. The studies I review here have all focused on the conditional reasoning paradigm. Inexplicably, some studies on conditional reasoning using ERPs have focused on contentless, abstract material (Bonnefond and Van der Henst, 2009). This is despite the fact that in the psychology of conditional inference, the dominant paradigm since Cummins et al. (1991) ground breaking paper has been the causal conditional inference task, which has arguably completely altered the theoretical landscape of research into the conditional.

The failure to consider the full theoretical possibilities is repeated in Bonnefond and Van der Henst (2013), who introduce the paper using the theoretical framework of mental logic, which has not been applied to any of the major results in conditional inference over the last twenty years of research. They argue that a sustained late positive component to the EEG waveform suggests that “participants consider logical arguments as a rule-governed sequence.” The absence of an N400 (a negative going waveform at around 400 ms) associated with semantic processing is not consistent with apparent inconsistencies being semantic in origin rather than formal. The implication of their results is that, even though their materials introduced content, the main effect was to facilitate activation of terms expected as a matter of logical inference.

However, even more recently Bonnefond et al. (2014) investigated the correlates of defeaters in conditional inference. A defeater in a causal conditional reasoning task is an event that could prevent the cause from producing its effect. For example, *if you turn the key the car starts*, is defeated by the *petrol tank being empty* or the *battery being flat*. In the Cummins paradigm, causal conditionals are pretested for the number of defeaters they allow. The primary behavioral observation is that the more defeaters a conditional allows, the less willing participants are to endorse the MP inference. Bonnefond et al. (2014) replicated these results and found specific effects on EEG waveforms. Their main finding was that presenting the conclusion of an MP inference led to,

“…a more pronounced N2 and less pronounced P3b for many disabler conditionals. In the ERP literature this specific N2/P3b pattern has been linked to the violation and satisfaction of expectations, respectively…Thereby, the present ERP findings support the idea that disabler retrieval specifically modulates our expectations that the standard MP conclusion will follow.” (Bonnefond et al. (2014), p. 258).

It is suggested that these results are consistent with conditional inference not being mediated by formal logical rules. Indeed the first demonstration of defeater effects (Byrne, 1989) was interpreted as refuting mental logicians’ explanation of why introducing alternative causes leads to reduced levels of the affirming the consequent fallacy (Braine et al., 1984). Such pragmatic factors may influence fallacies but they would not be expected to affect logical rules of inference such as MP if they play a role in real human inference. That is, Bonnefond et al.’s (2014) results showing the brain correlates of defeater information supplies just the evidence required to refute their interpretation of their own previous results (Bonnefond and Van der Henst, 2013). These results are also consistent with the probabilistic approach adopted in the new paradigm (Oaksford et al., 2000; Oaksford and Chater, 2007).

Another recent ERP study of conditional reasoning using the MP inference has shown a strong N400 component, which Bonnefond and Van der Henst (2013) did not observe (Blanchette and El-Deredy, 2014). This component of time locked EEG signals is strongly related to the processing of semantic content (Kutas and Hilyard, 1980). This early response to the premises of an argument is consistent with Kahneman’s banana-vomit example: the content of the premises is processed very rapidly. Blanchette and El-Deredy (2014) conclude that “conditional reasoning is not a purely formal process but that it importantly implicates semantic processing.” This conclusion is consistent with rapid System 1 processes which generate the kinds of information we discussed earlier and perhaps build an initial concrete model of the described situation. Of course this interpretation does not preclude System 2 involvement at some later point in the process.

In summary, the last two ERP studies reviewed are the closest to seeing System 1 in action. Bonnefond et al. (2014) also very commendably concede that their results question their earlier interpretation of their findings using abstract materials. Nonetheless, it is concerning that imaging results are published which do not consider the current state of theoretical development a topic has achieved in other areas of cognitive science. I can but agree with Bonnefond et al.’s (2014, p. 260) conclusion:

“Behavioral studies have also focused on the impact of different types of conditionals (e.g., tips, warnings, promises, and causal statements)…We belief [*sic*] that the present study will pave the way for a further exploration of the neural basis of these content factors in future studies.”

Such studies are a pressing need in this area but also required are methods that allow a much tighter integration between formal computational level theories of function and the brain.

## Conclusion

In this paper, I have discussed the interpretation of what is currently known about the brain systems involved in human deductive reasoning mainly using different imaging techniques to localize function to specific brain regions. In doing so, I have dealt with the results of Prado et al.’s (2011) meta-analysis and a range of other results from the perspective of the new paradigm in human reasoning. Prado et al. (2011) identified a relatively restricted group of brain regions consistently activated in deductive reasoning tasks. Like the studies in the meta-analysis, Prado et al. (2011) interpret their results largely in terms of mental logic and mental models theories. In this paper, I have reinterpreted most of these findings in terms of the new paradigm in reasoning which is a probabilistic dual process theory.

The first substantive issue to emerge was that Prado et al. identify their core left lateralized system with Gazzaniga’s left brain interpreter hypothesis. This identification is not consistent with this system being dedicated to deductive reasoning. The kinds of inferences that motivates Gazzaniga’s hypothesis are elaborative, defeasible inferences of the type that motivated the introduction of the probabilistic approach to human reasoning (Oaksford and Chater, 1991). Moreover, this is exactly the mode of inference, i.e., non-demonstrative inference involving world knowledge, which Fodor (1983) identified with central cognitive processes, i.e., those processes least likely to be subserved by an isolable cognitive module.

The second substantive issue concerned the apparent inability of the studies used in the meta-analysis to uncover the brain regions involved in System 1 processes. These are highly content dependent and are responsible for the automatic computation of a range of information used in inference. As I argued, the subtraction methodology and meta-analytic approach meant the whole brain diffuse activations caused by specific contents (Pereira et al., 2011) must have been subtracted out. Thus the current methodology would appear to leave us largely ignorant of the brain systems involved in System 1. I also explored a range of other results using different imaging techniques and two recent ERP studies (Blanchette and El-Deredy, 2014; Bonnefond et al., 2014) seem to show results capable of illuminating the nature of System 1.

Many of the studies using other techniques also seemed to have problems related to the third substantive issue concerning the interpretation of active brain regions. The interpretation of these findings depends on the computational level theory of the function engaged by a cognitive task. In general, either the attribution of function provided by Prado et al. (2011) was broadly consistent with the new paradigm, e.g., categorical reasoning, or it was clear that there were multiple interpretations of the function a region computed, e.g., abstract rules vs. similarity and analogy or small scale models of specific relations. Similar problems arose for the interpretation of studies of matching bias using fMRI (Prado and Noveck, 2006, 2007) and the NIRS studies of Tsujii et al. There is a failure to consider the full range of computational level interpretations available in the area.

While the localization approach has provided useful information about the brain systems involved in deductive reasoning, and its extension to looking a functional connectivity may be even more revealing, the interpretation of these results remains problematic. Certainly, following Goel (2007), I doubt that any single isolable region “does” deductive or inductive reasoning. Reasoning and inference are not special purpose add-ons to the cognitive system. Unconscious inference in perception and action, elaborative inference in language understanding and explicit verbal reasoning are major functions of the brain. These processes allow us to act adaptively and comprehend an uncertain world the state of which at any point in time we are mostly ignorant. Inference allows us to make the best guess about what will happen next, what someone means, and whether what they said is a good argument. One would imagine that a large amount of cortex would be dedicated to these processes.

System 1 automatically generates a large range of information and if the results using simple stimuli, e.g., thinking of horses, is anything to go by many diffuse brain regions will be activated by the materials in a reasoning problem. It is a reasonable hypothesis that this is the source of information for the left brain interpreter. The nature of System 2 is less clear. Results on logical intuitions suggest that people are unconsciously generating the logically correct answer even as they give the biased response. A radical possibility is that analytic (putatively System 2) and heuristic/probabilistic process (putatively System 1) are both computed by the one System, i.e., System 1 (Oaksford, 2014, Submitted). That is, in spontaneous human reasoning, without logical training, pencil and paper, computer, or friends, there is no conscious analytic process akin to the mental arithmetic required to solve the bat and ball problem. That is, all spontaneous reasoning is unconscious (Lakoff and Johnson, 1999). System 2 is where the products of these processes are posted and decisions made about which response to go with and which response to inhibit (Oaksford, 2014, Submitted). This is the core system most likely identified in Prado et al.’s (2011) meta-analysis, and thus interpreted, it seems that fMRI and lesions studies have been most revealing of these slow System 2 processes.

An approach is required that can reveal how the interactions between Systems unfolds over time and how these different systems communicate with the process of forming a response. System 1 responds rapidly and as we have seen, two very recent EEG studies (Blanchette and El-Deredy, 2014; Bonnefond et al., 2014), with good temporal resolution seem to provide the most informative studies of System 1 in action. Perhaps the most important innovation would be to conduct studies that had the potential to tightly correlate formal computational models of reasoning to brain activation be it using ERPs or fMRI. Many models of reasoning are formally well specified. These tend to be mostly emanating from the probabilistic side of the new paradigm (Oaksford and Chater, 1994; Chater and Oaksford, 1999; Oaksford et al., 2000). Formal models of dual processes are less in evidence, although Klauer et al. (2010), for example, present a formal model with a specific parameter that indexes System 1 vs. System 2 involvement. The value of such formal models is that model based imaging can reveal correlations between specific parameters of the formal model and brain activation providing much tighter integration between imaging results and the computational level. Pursing this line, I would argue, could provide a more integrated approach bringing the computational level and the implementational level into closer alignment.

## Conflict of interest statement

The author declares that the research was conducted in the absence of any commercial or financial relationships that could be construed as a potential conflict of interest.

## Footnotes

^1^This can be disputed (Schroyens, 2010). It is possible that mental models has introduced slippage between the computational and algorithmic levels. That is, mental models has been making advances by proposing a particular representation/process pair which can mimic logic under certain circumstances but the actual full computational level theory of mental models, i.e., the actual logic it implements at the algorithmic level, remains to be defined. This is a coherent proposal and there may be candidate logics that might make good on this claim. However, I have never heard this argument put forward by any other mental models theorist.

^2^The philosopher, Willard Van Ormond Quine, famously commented that a belief can always be saved from refutation by making adjustments elsewhere in our belief system, i.e., the mechanisms of belief fixation and revision are holistic, depending on everything else that we know or believe (Quine, 1953).

^3^This is also important because it suggests a unified account of System 1 and unconscious inference in perception and action (Oaksford, 2014, Submitted).

^4^Clearly the weight of evidence matters here. For example, if across a broad range of different tasks, t_1_…t_n_, thought to engage probabilistic reasoning, r_1_ is consistently activated but it is not in say t_n+1_, i.e., a nominally deductive task, then we might be begin to be persuaded that probabilistic reasoning is not involved in deductive tasks. However, (i) this question has not been investigated with a broad range of different tasks, and (ii) as we have argued that the bulk of probabilistic reasoning is a System 1 process, i.e., a central process unlikely to be associated with a single isolable brain region.

^5^De Neys et al. (2008) have shown that the anterior cingulate cortex, associated with conflict detection, is active when these two systems conflict.
